# Reading performance is predicted by more than phonological processing

**DOI:** 10.3389/fpsyg.2014.00960

**Published:** 2014-09-19

**Authors:** Michelle Y. Kibby, Sylvia E. Lee, Sarah M. Dyer

**Affiliations:** ^1^Department of Psychology, Southern Illinois University, Carbondale, ILUSA; ^2^Center for Integrated Research in Cognitive and Neural Sciences, Southern Illinois University, Carbondale and Springfield, ILUSA

**Keywords:** children, phonological awareness, rapid automatized naming (RAN), phonological memory, working memory, attention control, reading, word recognition

## Abstract

We compared three phonological processing components (phonological awareness, rapid automatized naming and phonological memory), verbal working memory, and attention control in terms of how well they predict the various aspects of reading: word recognition, pseudoword decoding, fluency and comprehension, in a mixed sample of 182 children ages 8–12 years. Participants displayed a wide range of reading ability and attention control. Multiple regression was used to determine how well the phonological processing components, verbal working memory, and attention control predict reading performance. All equations were highly significant. Phonological memory predicted word identification and decoding. In addition, phonological awareness and rapid automatized naming predicted every aspect of reading assessed, supporting the notion that phonological processing is a core contributor to reading ability. Nonetheless, phonological processing was not the only predictor of reading performance. Verbal working memory predicted fluency, decoding and comprehension, and attention control predicted fluency. Based upon our results, when using Baddeley’s model of working memory it appears that the phonological loop contributes to basic reading ability, whereas the central executive contributes to fluency and comprehension, along with decoding. Attention control was of interest as some children with ADHD have poor reading ability even if it is not sufficiently impaired to warrant diagnosis. Our finding that attention control predicts reading fluency is consistent with prior research which showed sustained attention plays a role in fluency. Taken together, our results suggest that reading is a highly complex skill that entails more than phonological processing to perform well.

## INTRODUCTION

Several researchers have supported the view that phonological processing is the “core” deficit in developmental dyslexia (for a review, see Snowling, 2000; Ramus, 2003). Consistent with this notion, prior researchers have demonstrated that phonological processing plays a role in multiple aspects of reading including basic reading, fluency, and comprehension (Cornwall, 1992; Wagner et al., 1993; Sprugevica and Hoien, 2004; Nelson et al., 2012). Three of the main components of phonological processing are phonological awareness, rapid automatized naming, and phonological memory (Wagner et al., 1993). Phonological awareness includes one’s ability to process and manipulate individual phonemes; rapid naming includes one’s ability to retrieve phonemes rapidly/automatically from long-term memory; and phonological memory refers to short-term memory for phonetically coded material. Research by Nelson et al. (2012) supports the idea that these skills are best conceived as three separate but correlated abilities rather than highly overlapping measures of one or two underlying constructs. Consistent with the idea of these being separate skills, these three abilities may differentially predict reading ability. Basic reading skills may have stronger associations with phonological awareness than with rapid naming (Wagner et al., 1997; Nelson et al., 2012), and reading fluency and comprehension may have stronger associations with rapid naming than with phonological awareness (Young and Bowers, 1995; Sprugevica and Hoien, 2004). However, these relationships may vary with the age/grade level of the reader (Wagner et al., 1993, 1997; Torgesen, 1999; Kirby et al., 2003). In their review, Wolf and Bowers (1999) suggested that phonological awareness is important for decoding, whereas rapid naming is important for reading fluency. Both skills may contribute to word identification and reading comprehension. Phonological memory may play a role in basic reading as well, especially decoding (Gathercole et al., 1991; Kibby, 2009). Phonological memory may play a greater role in basic reading than in fluency (Puolakanaho et al., 2008) or in comprehension (Kibby and Cohen, 2008).

Working memory (WM) may contribute to reading ability in addition to phonological processing. For example, Swanson repeatedly has found that WM plays a role in reading comprehension regardless of whether verbal or visual WM was assessed (for a review, see Swanson et al., 2009). Others also have shown that WM is related to reading ability (Baddeley et al., 1985; Vellutino et al., 2004; Kibby and Cohen, 2008; Sesma et al., 2009; Christopher et al., 2012). Moreover, when studying children with Attention-deficit/hyperactivity disorder (ADHD), Miller et al. (2013) found that poor WM contributes to their comprehension problems in that WM mediated the relationship between ADHD symptoms and the ability to recall the central ideas of the passage. WM may play a role in reading fluency as well (Baddeley et al., 1985). For example, Christopher et al. (2012) found WM to be related to word reading when a composite of word reading was used that included a timed measure. Furthermore, deficits in WM may play a role in the inefficient reading fluency often found in children with ADHD (Jacobson et al., 2011).

Attention-deficit/hyperactivity disorder frequently co-occurs with reading disability (RD) at a rate of 15–35% (Shaywitz et al., 1995). Although basic reading skills tend to be relatively preserved in children with ADHD alone, attention problems can impact their reading performance, even if at a subclinical level (Ghelani et al., 2004; Cain and Bignell, 2014). This is particularly true of reading fluency and comprehension. A possible contributor to the reduced reading performance in this group is poor sustained attention. Sustained attention may be related to both reading fluency and comprehension (Stern and Shalev, 2013). Consistent with this notion, lapses in attention as measured by reaction time variability have been shown to be predictive of fluency and comprehension (Jacobson et al., 2013; Tamm et al., 2014).

One area that was found to be lacking in the literature is the integration of these various predictors. More specifically, the relative contributions of phonological awareness, rapid automatized naming, phonological short-term memory, verbal WM, and attention control to the various aspects of reading is unknown. Therefore, our study compared these possible contributors in terms of how well they predict the various aspects of reading: word recognition, decoding, fluency and comprehension, in a mixed sample of children. We used a mixed sample in order to have a wide range of ability levels represented in both the independent and dependent variables. Furthermore, as all variables utilized vary on a continuum rather than being categorical in nature, looking at them from a continuous perspective is justified.

Based upon our review of the literature we hypothesized that phonological awareness would predict basic reading skills and reading comprehension; rapid naming would predict every reading skill except decoding; phonological memory would predict basic reading skills; and WM and attention control would predict reading fluency and comprehension.

## MATERIALS AND METHODS

### PARTICIPANTS

Participants included 182 children, ages 8–12 years. Thirty had a reading disorder, 65 had ADHD, 35 had both disorders, and 52 were typically developing children as determined by a child neuropsychologist. Group membership is provided for descriptive purposes, since groups were not compared in this study for the reasons noted above. Children with other psychiatric, neurological, or medical diagnoses were excluded from this study. Additional exclusion criteria included significant pre- or post-natal complications, suspected abuse, and an IQ below 80.

The participants are from a community sample. Parents brought them to the first author’s laboratory for the study. The children attended various schools in our region, including public and private schools. Most of the children with reading problems have a history of intervention such as special education services, remedial services, and/or tutoring. Some of the children with ADHD were previously diagnosed and treated with stimulant medication, but none were on medication at the time of testing.

### MEASURES

#### Phonological processing

Phonological awareness and rapid automatized naming were measured using Elision and Rapid Letter Naming (RLN) from the Children’s Test of Phonological Processing (CTOPP; Wagner et al., 1999). Elision is considered to be a measure of phonological awareness, as it requires both analysis and synthesis of phonemes. The child must remove the stated phoneme from a word and blend the remaining phonemes to form a new word. It is an orally administered subtest. RLN requires that the child name a series of printed letters as quickly as possible. Internal consistency for Elision ranges from 0.86 to 0.91 for 8- to 12-year-old children. Since RLN is a timed measure, alternate-form reliability was used instead of internal consistency and ranged from 0.73 to 0.87 for 8- to 12-year-old children (Wagner et al., 1999). The CTOPP has good validity as well (Mitchell, 2001; Haight, 2006).

#### Memory

Phonological short-term memory and verbal WM were measured using Digit Span Forward (DSF) and Digit Span Backward (DSB) from the Wechsler Intelligence Scale for Children-Fourth Edition (WISC-IV; Wechsler, 2003). DSF requires immediate, verbatim recall of lists of digits presented at one digit per second. DSB requires the child to immediately recall lists of digits in reverse order. We used the WISC-IV to determine the participants’ IQ as well. The reliability and validity of the WISC-IV have been well-established according to the manual, with internal consistency of 0.83 and 0.80 for DSF and DSB, respectively.

#### Attention

Effectiveness of attention control was measured by parent-report using the Behavior Assessment System for Children – Second Edition (BASC-2; Reynolds and Kamphaus, 2004). The age-appropriate form (child form for ages 8–11 and adolescent form for age 12) was used with gender-specific norms. The Attention Problems scale on the BASC-2 measures common symptoms of inattention such as “has a short attention span”, “pays attention when spoken to,” and “is easily distracted.” The parent-report form of the BASC-2 has good reliability and validity according to the manual, with Attention Problems having an internal consistency of 0.87 for the child form and 0.88 for the adolescent form.

#### Reading ability

Reading was measured with the Woodcock–Johnson Tests of Achievement – Third Edition (WJ-III Form A; Woodcock et al., 2001). Decoding was measured using Word Attack, which requires the child to decode pronounceable non-words. Word identification was measured using the Letter-Word Identification subtest, which requires the child to identify words of increasing difficulty at our age range (8–12). Reading comprehension was measured using the Passage Comprehension subtest, which requires the child to read sentences or paragraphs and provide an appropriate word to complete the passage. Reading fluency was measured using the Reading Fluency subtest, which requires the child to read a series of brief statements quickly and determine if each statement is true or false. Scoring on this subtest is based on the number of accurate responses within a 3-min time limit. It was the only timed measure of reading used. Median internal consistency for these four subtests ranges from 0.87 and 0.94 (Schrank et al., 2001). Generally, the Woodcock–Johnson Tests of Achievement has well-established reliability and validity according to the test’s technical manual.

### PROCEDURES

All participants partook in a full day of testing, including the measures above, as part of a larger project. Participants’ parents completed questionnaires on the participants and took part in an interview designed to obtain background information on their child. The measures selected for the project were chosen in order to assess multiple areas of functioning. For time reasons, complete test batteries, such as the WJ-III and CTOPP, were not administered. Rather, the subtests that were believed to best represent the constructs of interest were selected.

The project from which this study was derived was approved by the Southern Illinois University Institutional Review Board’s Human Subjects Committee and conformed to all relevant regulatory standards. All participants provided informed assent, and their parent/legal guardian provided informed consent before testing began.

## RESULTS

### PRELIMINARY ANALYSES

All variables were checked for normality and found to be normally distributed. **Table 1** includes the demographic and descriptive data for our sample. Of note, the sample as a whole tended to perform in the Average range on the various measures, with distributions in the expected range based on the various tests’ means and standard deviations.

**Table 1:** Demographic and descriptive data.

Variable	
Gender	53.7% male
Race/Ethnicity	93.1% Caucasian
Maternal Education Level	31.7% Bachelor’s degree

**Variable**	**Mean**	**Standard deviation**

Age (years)	9.55	1.36
CTOPP Elision	93.82	15.78
CTOPP Rapid Letter Naming	92.96	12.52
WISC-IV Digit Span Forward	8.48	2.57
WISC-IV Digit Span Backward	8.98	2.83
WJ-III Letter Word Identification	95.21	15.17
WJ-III Word Attack	97.08	11.98
WJ-III Reading Fluency	92.80	16.69
WJ-III Passage Comprehension	92.98	12.90
BASC-2 Attention Problems	59.08	11.17

Most measures have a mean of 100 and a SD of 15 except for Attention Problems, which has a mean of 50 and a SD of 10, and Digit Span, which has a mean of 10 and a SD of 3.

### MAIN RESULTS

**FIGURE 1:**
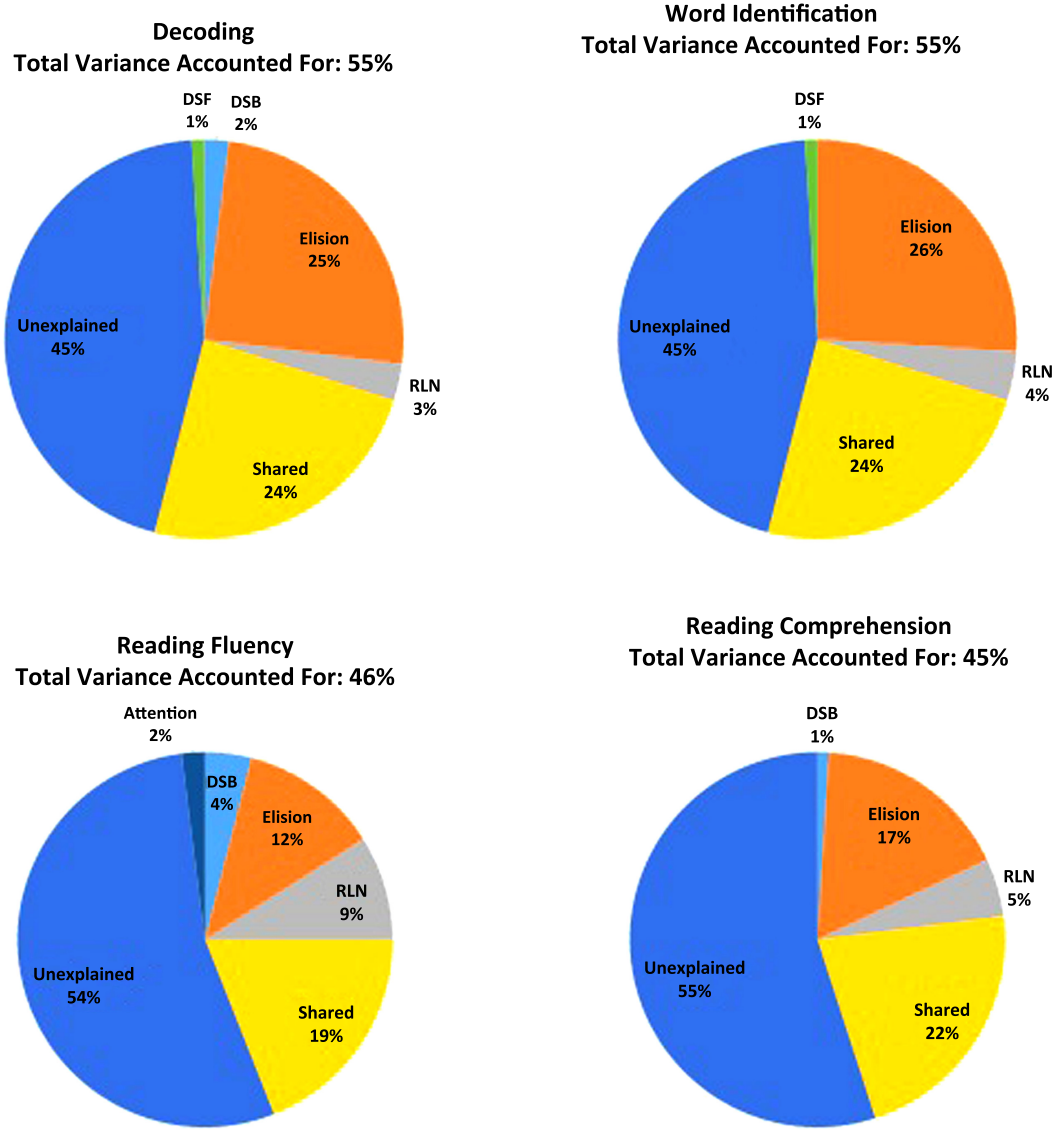
**Percentage of the total variance explained by Elision, Rapid Letter Naming (RLN), Digit Span Forward (DSF), Digit Span Backward (DSB), and Attention Problems (Attention) in basic reading, reading fluency and reading comprehension**.

To assess our hypotheses, we conducted four multiple linear regressions using SPSS in which Elision, RLN, DSF, DSB, and Attention Problems were entered to predict decoding, word recognition, reading fluency and reading comprehension, respectively. Our findings are summarized in **Figure 1** and **Table 2**.

**Table 2:** Predictors of reading ability in β units.

	Aspects of reading			
Variable	Word identification	Decoding	Fluency	Comprehension
CTOPP Elision	0.57***	0.56***	0.38***	0.46***
CTOPP Rapid Letter Naming	0.23***	0.16**	0.31***	0.24***
WISC-IV Digit Span Forward	0.12*	0.13*	0.02	0.11
WISC-IV Digit Span Backward	0.08	0.14**	0.23***	0.13*
BASC-2 Attention Problems	-0.04	0.002	-0.14*	-0.06

*p < 0.05, **p < 0.01, ***p < 0.001.

Our results revealed that all of the phonological processing components, as well as DSB, significantly predicted decoding performance, *F*(5,165) = 42.85, *p* < 0.001. More specifically, Elision [*t*(165) = 9.79, *p* < 0.001], RLN [*t*(165) = 2.93, *p* = 0.004], DSB [*t*(165) = 2.55, *p* = 0.01], and DSF [*t*(165) = 2.36, *p* = 0.02] all positively predicted performance. However, Attention Problems did not predict this basic reading skill [*t*(165) = 0.05, *p* = 0.96]. Together the predictor variables accounted for 55% of the variance in decoding performance when using adjusted R^2^.

The combination of independent variables significantly predicted word identification as well, *F*(5,165) = 43.00, *p* < 0.001 accounting for 55% of the variance when using adjusted R^2^. Elision [*t*(165) = 9.82, *p* < 0.001], RLN [*t*(165) = 4.17, *p* < 0.001], and DSF [*t*(165) = 2.18, *p* = 0.03] were significant predictors, while DSB [*t*(165) = 1.48, *p* = 0.14] and Attention Problems [*t*(165) = -0.68, *p* = 0.50] were not.

The regression predicting reading fluency was significant also, *F*(5,158) = 28.27, *p* < 0.001, explaining 46% of the variance when using adjusted R^2^. Furthermore, all of the independent variables were significant except DSF [*t*(158) = 0.34, *p* = 0.74]. More specifically, Elision [*t*(158) = 6.02, *p* < 0.001], RLN [*t*(158) = 5.23, *p* < 0.001], and DSB [*t*(158) = 3.63, *p* < 0.001] were all positive predictors of reading fluency. Attention Problems had a negative relationship with fluency [*t*(158) = -2.32, *p* = 0.02].

Finally, the regression predicting passage comprehension was significant, *F*(5,164) = 28.85, *p* < 0.001, accounting for 45% of the variance when using adjusted R^2^. Similar to reading fluency, passage comprehension was not significantly predicted by DSF, *t*(164) = 1.78, *p* = 0.08. Attention Problems also was not a significant predictor, *t*(164) = -1.01, *p* = 0.32. However, Elision [*t*(164) = 7.19, *p* < 0.001], RLN [*t*(164) = 3.93, *p* < 0.001], and DSB [*t*(164) = 2.13, *p* = 0.04] all positively predicted reading comprehension performance.

## DISCUSSION

Taken together, our findings support the notion that phonological processing is a core contributor to reading ability regardless of the aspect of reading being assessed. Nonetheless, we found that phonological processing was not the only predictor of reading performance. Verbal WM and attention control predicted reading ability as well, at least for some aspects of reading performance. Thus, reading appears to be a highly complex skill, requiring multiple abilities to perform it well.

When breaking down phonological processing into its component skills, we hypothesized that phonological awareness would predict basic reading skills and reading comprehension based on the work of Wagner et al. (1997) and the review by Wolf and Bowers (1999). We found this to be the case. In addition, when looking at its relative contributions to the various reading skills, phonological awareness was a better predictor of basic reading skills than reading fluency or comprehension, consistent with the work of Gray and McCutchen (2006). Nonetheless, phonological awareness also predicted reading fluency, which we did not hypothesize. In fact, phonological awareness was the best single predictor of every reading skill assessed. Thus, even among the phonological processing components it may be a “core” skill.

Another important contributor to reading performance is rapid automatized naming (Wagner et al., 1997; Wolf and Bowers, 1999). We hypothesized that rapid naming would predict every reading skill assessed except decoding. Our findings were generally consistent with this hypothesis except that we also found rapid naming to predict decoding. In fact, RLN was the second best predictor of every reading skill assessed, accounting for 3–9% of the explained variance. Consistent with the review by Wolf and Bowers (1999), RLN was a better predictor of reading fluency than it was of the other reading skills. However, in contrast to this review and the work of Sprugevica and Hoien (2004), rapid naming was a close second to phonological awareness in its ability to predict reading fluency, suggesting both skills may be of comparable importance in our age range (8–12 years).

In terms of the third component of phonological processing, we hypothesized that phonological memory would predict basic reading skills. Our findings are consistent with this hypothesis, as phonological memory was a significant predictor of both basic reading skills. Nonetheless, it only explained a small portion of the variance when in equations with phonological awareness and RLN. In general, our findings on phonological memory are consistent with prior research showing that phonological short-term memory is a contributor to basic reading skills (Gathercole et al., 1991; Kibby, 2009). It has been proposed that phonemes are held in phonological short-term memory during segmentation and blending when forming words (Snowling, 2000).

It has been debated whether these three tests of phonological processing (phonological awareness, rapid automatized naming, and phonological memory) are measuring separate but correlated abilities or whether they are highly overlapping measures of one or two underlying constructs. Whereas research by Nelson et al. (2012) supports the idea that these tests are best conceived as measuring separate but correlated abilities, research by Wagner and colleagues (1987, 1993) suggests that these tests may be best construed as measures of one or two underlying constructs. Our findings are consistent with the former position in that all three tests predicted at least some aspects of reading ability despite being entered into the same equations. In addition, Pearson correlations between the three tests were significant at the 0.001 alpha level but were moderate, ranging from 0.2 to 0.4.

Another important contributor to reading performance beyond phonological processing is WM (Vellutino et al., 2004; Kibby and Cohen, 2008; Sesma et al., 2009; Christopher et al., 2012). WM may be particularly important for reading comprehension (for a review, see Swanson et al., 2009) and reading fluency (Baddeley et al., 1985). Based on this literature, we hypothesized that WM would predict both reading fluency and comprehension. This hypothesis was supported in that our measure of verbal WM accounted for a unique portion of the variance in both reading skills. However, verbal WM also predicted decoding skill beyond phonological memory, which suggests that it may support the mental operations of analysis and synthesis that are required to decode novel items. Thus, when utilizing Baddeley’s model of WM (Baddeley, 2007), it appears that the phonological loop contributes to basic reading ability and the central executive contributes to reading fluency and comprehension, along with decoding skill. Consistent with the work of Jacobson et al. (2011) which showed WM is important for efficient reading in a sample with ADHD, the central executive explained the greatest proportion of variance in fluency of all the reading skills, explaining 4% of the variance.

We hypothesized that attention control would predict both reading fluency and comprehension based on the prior literature, but we found that it only predicted fluency. Thus, inattention may play a more limited role in basic reading skills. This statement is commensurate with the finding that over half of children with ADHD do not have a comorbid learning disability in basic reading (Shaywitz et al., 1995). We anticipated that attention control would affect reading fluency as inattention can result in both errors and slowed rate intuitively. This supposition and our results are consistent with the work of Stern and Shalev (2013) who found poor sustained attention to be associated with longer reading duration. It also is consistent with the work of Jacobson et al. (2013) who found lapses in attention to be predictive of worse reading fluency. Because our reading comprehension measure only included about one paragraph per item, it is unknown whether inattention would affect comprehension of a longer passage, such as that found in most texts a child would read. Hence, this aspect of reading comprehension is worthy of future study.

Our findings in total suggest that (1) phonological processing, particularly phonological awareness and rapid automatized naming, are core processes involved in reading, and (2) other cognitive processes contribute to reading ability as well, with the proportion of variance explained being variable across reading measures. Therefore, our results are generally commensurate with the phonological-core variable-difference model put forth by Stanovich (1988). He suggested that most students with a RD involving word recognition have a core deficit in phonological processing. However, “garden variety” poor readers have additional deficits in other cognitive skills that vary across individuals. This variability was believed to contribute to the individual differences seen across poor readers. Although we studied the continuum of reading ability from impaired to superior rather than just poor readers, we did find that phonological processing was the primary predictor of each aspect of reading assessed, being a core contributor, and that WM and attention control explained additional variance in some reading skills. Our findings also are generally commensurate with the work of Morris et al. (1998) who found that children with RD usually have impaired phonological awareness, with more variability in deficits being found across individuals in rapid naming, phonological memory, and other cognitive skills. Although we did not study children with RD specifically, we did find that phonological awareness accounted for the greatest proportion of variance in reading skill across the reading measures used, and this was most pronounced in basic reading skills.

Although our study may be the first to compare multiple phonological processing skills, verbal WM, and attention control in terms of how well they predict the various reading abilities, it has several limitations. One limitation is that this sample was only tested once, so we cannot compare the various predictors over time. It is quite possible that rapid naming and other cognitive skills (WM, attention control) could play a larger role in reading ability if we assessed older children/adolescents. This is because various researchers have found that predictors of reading ability vary across development (Wagner et al., 1993; Torgesen, 1999; Kirby et al., 2003), with phonological awareness being particularly important in the younger grades. Using a longitudinal format would help researchers understand how these predictors change and interact over the course of development. Another limitation is that our measures of reading fluency and comprehension utilized short sentences and passages, respectively. The predictors of fluency and comprehension could vary if longer texts were used, with WM and attention problems being expected to play a greater role. Third, our measure of attention control was from a parent-rated questionnaire. Our results would be clearer as to whether poor sustained attention and/or other aspects of inattention were driving this result if experimental measures were used. Fourth, we included a wide range of reading ability in our sample. Thus, future research should investigate the independent variables we used in terms of how well they predict reading functioning in different clinical groups (e.g., learning disability in word recognition, learning disability in reading comprehension, ADHD) to determine if there are differences in predictors amongst the clinical groups and controls.

## AUTHOR CONTRIBUTIONS

Michelle Y. Kibby worked with Sylvia E. Lee and Sarah M. Dyer to develop the idea for this manuscript. Michelle Y. Kibby is the PI on this project and did most of the writing of this manuscript. Sylvia E. Lee conducted the analyses and wrote the Results section including **Table 1** and the figure. Sarah M. Dyer wrote the Methods section. All three individuals worked on the literature review, reference section and editing of the manuscript.

## Conflict of Interest Statement

The authors declare that the research was conducted in the absence of any commercial or financial relationships that could be construed as a potential conflict of interest.
